# 16S rRNA Gene Amplicon Sequencing Data of Bacterial Community of Freshwater Sponge Lubomirskia baicalensis

**DOI:** 10.1128/mra.01164-21

**Published:** 2022-02-03

**Authors:** Sergei I. Belikov, Ivan S. Petrushin, Lubov I. Chernogor

**Affiliations:** a Limnological Institute, Siberian Branch of the Russian Academy of Sciences, Laboratory of Analytical and Bioorganic Chemistry, Irkutsk, Russia; Indiana University, Bloomington

## Abstract

Our study was devoted to investigating the mass disease and mortality of freshwater sponges (Lubomirskiidae) in Lake Baikal. The first sights of the disease were discovered in 2011 and were associated with a shift in sponge microbial diversity. To study the microbiome, we performed sequencing of the 16S rRNA amplicon DNA extracted from the freshwater sponges.

## ANNOUNCEMENT

Lake Baikal is the deepest (maximum depth, 1,642 m) and oldest freshwater lake (1, 2). Sponges endemic to Lake Baikal are filter-feeding invertebrates that contain a variety of symbiotic microorganisms. In recent years, there has been an increase in diseases and deaths of freshwater Baikal sponges in different areas of the lake. This mass disease endangers the biodiversity of the whole ecosystem of Lake Baikal. Disease of sponges is accompanied by a significant diversity shift in the sponge microbiome, including pathogenic microorganisms (3, 4).

To study the microbiome, we performed high-throughput sequencing of the 16S rRNA amplicon DNA extracted from the freshwater sponges. We collected 24 samples (6 healthy and 18 diseased) of the freshwater sponge species Lubomirskia baicalensis in the central region of Lake Baikal, Russia (53.097222 N, 107.438056 E) at depths of 12 to 15 m in 2019.

The V4 region of the 16S rRNA gene was amplified using modified primers 515F (5′-GTGYCAGCMGCCGCGGTAA-3′) and 806R (5′-GGACTACNVGGGTWTCTAAT-3′) and TransStart FastPfu DNA polymerase (TransGen Biotech, China) according to the manufacturer’s instructions (5). PCR amplification was performed using the following cycling conditions: 94°C for 5 min; 35 cycles of 94°C for 30 s, 55°C for 30 s, and 72°C for 1 min; and finally 72°C for 10 min. The resulting PCR products were examined by 2% agarose gel electrophoresis and further purified using a gel extraction kit (Omega Bio-tek, USA). The libraries were generated using PCR with Illumina adapters connected (New England BioLabs, USA) and were sequenced on the MiSeq platform (Illumina, USA).

The paired-end libraries were prepared using innuPREP forensic kit (Analytic Jena, Germany) according to the manufacturer’s protocol. Sequencing of the libraries was conducted with the 2 × 300-bp paired-end method on the MiSeq genome sequencer using a MiSeq reagent kit v3 at The Center of Shared Scientific Equipment “Persistence of microorganisms” (ICIS UB RAS, Russia).

Raw reads were preprocessed and filtered using QIIME 2 software (6) with the DADA2 plugin, the first 13 bp of reads were trimmed, and all reads were truncated to 250 bp. Alignment and taxonomic classification were performed according to the QIIME 2 protocol (7) with the SILVA reference database v. 138. A detailed description of samples is provided in Table 1.

**TABLE 1:** Summary description of sample data in this study

Sample no.	Library ID^*a*^	Sponge type	No. of raw sequencing reads	No. of reads, passed QC^*b*^	SRA accession no.	BioSample accession no.
64	1718	Diseased	267,280	143,214	SRR17073767	SAMN23515232
65	1719	Healthy	168,048	84,992	SRR17073766	SAMN23515233
66	1720	Diseased	157,142	81,904	SRR17073755	SAMN23515234
67	1721	Diseased	375,158	195,976	SRR17073748	SAMN23515235
68	1726	Diseased	453,724	222,626	SRR17073747	SAMN23515236
69	1727	Diseased	170,900	91,308	SRR17073746	SAMN23515237
70	1733	Diseased	133,018	68,946	SRR17073745	SAMN23515238
71	1734	Healthy	141,542	73,056	SRR17073744	SAMN23515239
72	1735	Healthy	131,676	67,132	SRR17073743	SAMN23515240
73	1836	Diseased	129,296	66,630	SRR17073742	SAMN23515241
74	1837	Diseased	126,554	70,386	SRR17073765	SAMN23515242
75	1738	Healthy	161,294	86,774	SRR17073764	SAMN23515243
76	1743	Healthy	130,466	66,460	SRR17073763	SAMN23515244
77	1744	Diseased	197,220	107,472	SRR17073762	SAMN23515245
78	1745	Diseased	138,840	75,016	SRR17073761	SAMN23515246
79	1746	Diseased	141,542	76,068	SRR17073760	SAMN23515247
80	1747	Healthy	141,322	65,932	SRR17073759	SAMN23515248
81	1781	Diseased	129,862	68,964	SRR17073758	SAMN23515249
82	1783	Diseased	113,052	61,608	SRR17073757	SAMN23515250
83	1789	Diseased	152,332	81,330	SRR17073756	SAMN23515251
84	1790	Diseased	120,648	62,886	SRR17073754	SAMN23515252
85	1791	Diseased	183,218	97,816	SRR17073753	SAMN23515253
86	1792	Diseased	120,850	65,348	SRR17073752	SAMN23515254
87	1820	Diseased	100,932	54,696	SRR17073751	SAMN23515255
88	MQ	Negative control	636	106	SRR17073750	SAMN23515256
89	K+	Positive control	104,338	55,270	SRR17073749	SAMN23515257

a ID, identifier.

b QC, quality control.

**FIG 1:**
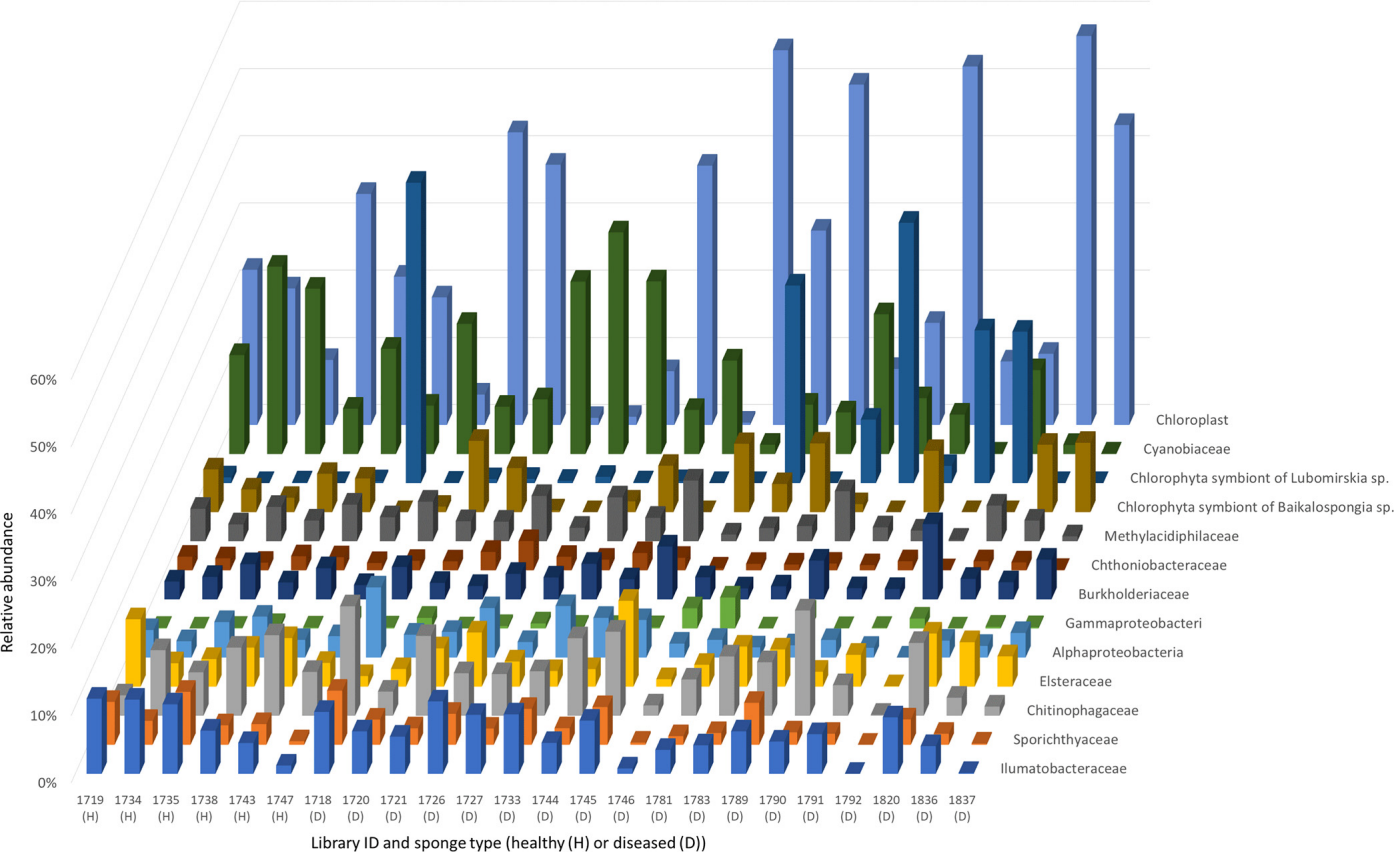
Microbial community composition in freshwater sponge samples.

The composition of the microbial community in healthy and diseased sponges is shown in Fig. 1, where the major taxa are chloroplasts, *Cyanobacteria*, *Chlorophyta* symbiont, *Verrucomicrobia*, several *Proteobacteria*, *Bacteroidetes*, and *Actinobacteria*. The difference between healthy and diseased sponge samples is unclear. Thus, we conclude that detecting the microbial differences between healthy and diseased sponge samples to the family level does not allow identifying primary pathogens. Further research requires deeper sequencing of the hologenomes of sponges or cultivation of the microorganisms on selective media.

Our study allows us to describe the taxonomic composition of the microbial communities in sponges of Lake Baikal, which makes it possible for future researchers to prepare more experiments for the isolation of putative pathogens of freshwater sponges.

### Data availability.

The raw sequence data have been deposited in the NCBI SRA repository via BioProject PRJNA784928 (BioSample and SRA accessions are provided in Table 1).
